# Farming exposures and Alzheimer's disease: cross-sectional analysis within the French AGRICAN cohort

**DOI:** 10.5271/sjweh.4284

**Published:** 2026-05-01

**Authors:** Victoire Madeline, Angéline Galvin, Lucie De Graaf, Julien Engelhardt, Pierre Lebailly, Isabelle Baldi

**Affiliations:** 1 Univ. Bordeaux, Inserm, Bordeaux Population Health Research Centre, ACTIVE team, UMR 1219, Bordeaux, France.; 2 Univ. Bordeaux, Inserm, Bordeaux Population Health Research Centre, EPICENE team, UMR 1219, Bordeaux, France.; 3 Service Santé Travail Environnement, Pôle de Santé Publique, CHU de Bordeaux, Bordeaux, France.; 4 Service de Neurochirurgie B, CHU de Bordeaux, Place Amélie Raba-Léon, Bordeaux Cédex, France.; 5 Univ. Bordeaux, Bordeaux INP, CNRS, IMB, UMR 5251, Talence, France.; 6 INSERM, UMR1086 ANTICIPE, Cancers et Préventions, Centre François Baclesse, Caen, France.; 7 Université Caen Normandie, Caen, France.

**Keywords:** agriculture, agricultural exposure, cognitive impairment, farmer, pesticide

## Abstract

**Objectives:**

There is epidemiological evidence of an association between occupational pesticide exposure and cognitive impairment, but studies on the link with Alzheimer's disease are scarce. We explored the association between agricultural exposures and Alzheimer's disease in the AGRICAN cohort.

**Methods:**

We analyzed the relationship between doctor-diagnosed Alzheimer's disease and life-long exposures separately among men and women with the following exposures: work on a farm, pesticide use in any job, growing specific crops (N=13) or rearing animals (N=5), pesticide use on these crops/livestock, with adjustment for age, education, smoking, alcohol consumption and body mass index.

**Results:**

Among 109 287 participants in the analysis, 818 (267 men, 551 women) were classified as Alzheimer's disease cases. Increased risks were seen for work on a farm [men: odds ratio (OR) 1.81, 95% confidence interval (CI) 0.92–3.57; women: OR 1.58, 95% CI 0.94–2.86] or pesticide use in any job (men: OR 1.14, 95% CI 0.85–1.53; women: OR 1.42, 95% CI 1.10–1.85). Risks for crops and livestock were close to unity when compared with non–farmers, except for pigs (OR 1.38, 95% CI 1.01–1.89) and rapeseed among men (OR 1.45, 95% CI 1.00–2.11) and sunflower among women (OR 1.55, 95% CI 0.90–2.66). Using pesticides increased the risk among men especially for sheep/goats (OR 1.98, 95% CI 1.18–3.34), pigs (OR 1.80, 95% CI 1.19–2.74), potatoes (OR 1.47, 95% CI 1.03–2.10) and meadows (OR 1.54, 95% CI 1.14–2.08). Among women, risks associated with pesticide use on crops were generally elevated, reaching a two–fold increase for corn, rapeseed, sunflower, field peas and fruit growing.

**Conclusion:**

Our results suggest that agricultural exposures may play a role in Alzheimer’s disease among both men and women, with the highest risks associated with pesticide use in certain livestock and crop activities.

In 2020, an estimated 55 million individuals across the world were living with dementia, a number that almost doubles every 20 years (1). Alzheimer's disease represents about 60 to 70% of all dementia cases (2). Recent estimates point to 36 million individuals with Alzheimer's disease worldwide (3), a number that reaches 426 million when we include individuals with prodromal or preclinical stages (4, 5). Several studies have shown a decrease in incidence over time in higher-income countries (6). Age is the main determinant of the disease, although nearly 10% of the cases occur before the age of 65 (7, 8). The other main risk factors are female gender, genetic susceptibility and family history (9, 10). Considerable efforts have been made to better understand modifiable risk factors (6). Evidence of an increased risk has been provided for some factors, such as low levels of education, smoking, midlife obesity, hypertension, alcohol consumption, diabetes, depression and physical and cognitive inactivity (10, 11). However, fewer studies have explored the role of environmental and occupational exposures, such as air pollution, electromagnetic fields, metals, and pesticides (12, 13). Fewer than fifteen studies have provided original data on the role of pesticides in the development of Alzheimer's disease. A review including six of them suggested that there was evidence of an association between occupational exposure to pesticides and Alzheimer's disease (12). A meta-analysis performed in 2016 added only one study and, from three cohorts and four case–control studies (6835 individuals including 1050 cases), calculated a combined odds ratio (OR) of 1.34 [95% confidence interval (CI) 1.08–1.67] with no significant heterogeneity (14).

Pesticides have been used widely in agriculture for decades, with annual use of around 2 000 000 tons worldwide (15). The French agricultural setting offers a variety of crops and livestock with a high pesticide use and an opportunity to identify risks related to specific crops/livestock (16). The present study is therefore based on AGRICAN, a large prospective cohort that aims to measure the association between agricultural exposures and Alzheimer's disease.

## Methods

### The AGRICAN cohort

The AGRICAN cohort enrolled active and retired workers in agriculture and related sectors in France starting in 2005, with the main objective of assessing the relationship between agricultural exposures and cancer incidence and mortality. A secondary aim related to neurological health. The eligible population consisted of all men and women, active or retired, affiliated with the French health insurance for farmers and workers in agriculture and related sectors (greenspaces, wood industry, cooperatives, tertiary sector…). Eligible individuals were those aged ≥18 years, affiliated for ≥3 years over their lifetime, and living in 2004 in one of the 11 French regions covered by a population-based cancer registry. A total of 181 842 individuals completed a self-administered enrolment questionnaire. Details of AGRICAN have been described previously (16), and questionnaires are available online (https://www.agrican.fr/pdf/questionnaires/questionnaire_AGRICAN_Inclusion_en.pdf).

The enrolment self-administered questionnaire included data regarding sociodemographic information, health history at enrolment (15 diseases including Alzheimer's disease, hypertension and diabetes), lifestyle habits (smoking, alcohol consumption…) and occupational data with a complete *curriculum laboris*, and the history of 13 crops (grassland, vineyard, wheat or/and barley, corn, field peas or/and beans, potato, tobacco, beet, sunflower, rapeseed, fruit-growing, greenhouses, vegetables) and five livestock (cattle, sheep/goats, pigs, horses, poultry), with years of start and end of production. This questionnaire also included questions on specific tasks related to crops and livestock, including pesticide use.

### Outcome definition

At enrolment, participants were asked whether a doctor had ever told them that they had Alzheimer's disease. If participants answered affirmatively, the age range at diagnosis was recorded (<20, 20–39, 40–60, >60 years). We also asked whether the questionnaire was completed with the help of – or by – a next of kin. In such cases, a participant was considered to have the disease if he/she or his/her relative had answered positively to the question about Alzheimer's disease or had mentioned an age at diagnosis. A participant was considered Alzheimer's-free if he/she responded in the negative. Participants with missing data on Alzheimer's disease or covariates were excluded from our analysis.

### Covariates

Analyses were stratified by gender and adjusted for the following risk factors described in the literature: age, education level (no school or primary level, secondary school, higher level), smoking (pack-years), alcohol (never, monthly, weekly, daily) and body mass index (BMI). In sensitivity analysis, we further adjusted on hypertension and diabetes.

### Assessment of exposure to crops, livestock and pesticides

We used four definitions to measure exposure to cover pesticide use in agriculture as well as outside agriculture, in any job throughout life, and also to consider proxies for other risk factors in agriculture. The first one was based on the complete job history that was translated into three groups of workers: farmers (report of working on a farm); agriculture-related workers (jobs outside farms in which pesticides could be used: eg, greenspaces, forestry, cooperatives); and non-agricultural workers (eg, craftsmen, fishermen, beekeepers, workers in the agricultural tertiary sector). The second definition was based on the type of livestock and crops grown throughout life (yes/no). The third definition was based on the participants' responses to questions regarding occupational use of pesticides (in agriculture or other industries). The question was as follows: “Have you ever used fungicides or insecticides or herbicides at work?” (yes/no).The last definition was based on items regarding use of pesticide on specific crops and livestock that they had grown or raised, with exposure quantified both as crop surface area or animal numbers and duration of pesticide use on crops and animals. We did not collect information on pesticide use on greenhouse crops or on vegetables, as the high diversity of crops grown within these productions made it difficult to obtain consistent exposure data. Additionally, for potatoes, no information was available on surfaces treated or duration of treatments. There was also a question on pesticide poisoning, phrased as follows: “Have you ever experienced pesticide poisoning?”.

### Statistical analyses

Logistic regression models were run to study the association between the disease and sociodemographic characteristics and the various exposures described above. All models were adjusted for age (continuous variable) and conducted separately according to gender considering that: (i) there were differences in the prevalence of Alzheimer's disease (male-to-female ratio = 2/3 in previous studies (1, 17–23); (ii) types of agricultural tasks vary according to gender; and (iii) different biological mechanisms could operate among men and women (20).

In the main analysis, the comparison group consisted of participants who never worked on a farm (subsequently referred to as “non-agricultural participants”). Sensitivity analyses were performed: (i) considering other farmers (not exposed to the studied livestock/crop) in the comparison group; (ii) removing diseased individuals who responded themselves; (iii) restricting patients with a diagnosis to ≥60 years old; and iv) further adjusting for hypertension and diabetes; and (iv) running analyses with 'age' variable transformed (logarithmic, quadratic, cubic splines) to see if this would better model the effect of exposure on Alzheimer's disease. In addition, dose–response relationships for quantitative exposures were explicitly analyzed in the models, as follows: for crops, per 10 hectares treated and per 10 years of pesticide use; for livestock, per 10 animals raised and per 10 years of insecticide use”. Multiple imputation was also performed for missing covariate data. Analyses were performed using Stata V17.0.

### Ethical approval and data protection

The AGRICAN study protocol was reviewed and approved by the Advisory Committee on Information Processing in Material Research in the Field of Health (*Comité Consultatif sur le Traitement de l′Information en matière de Recherche dans le domaine de la Santé*, number 01.148) and the French data protection authority (*Commission Nationale Informatique et Libertés*, number 05.1292). All study participants gave their informed consent by sending back the enrolment questionnaire.

## Results

Among the 181 842 participants, 149 251 completed questions on Alzheimer's disease, and among them 109 287 had complete information on all covariates (61 181 men and 48 106 women). In this sample, 818 individuals were classified as suffering from Alzheimer's disease (551 women and 267 men). Compared to these 818 patients, the non-included cases (N=679) were one year older (mean 81.9 versus 80.8 years), more frequently males (40.9 versus 32.6%) and smokers (30.7 versus 17.6%) but they had a similar level of education (85.6 versus 85.5% primary level), the same daily alcohol consumption (21.5 versus 23.5%) and BMI (25.0 versus 25.3 kg/m^2^)^.^ The frequency of work on a farm was also comparable (95.1 versus 96.4%).

The median age at enrolment was 60 and 64 years among men and women, respectively (table 1), higher in Alzheimer's disease cases (79 and 83 years among men and women, respectively). Participants without the disease predominantly had secondary level education among men (49.4%) and primary level among women (51.2%), while most of the Alzheimer's disease cases had primary level education (79.4% and 88.6% among men and women, respectively). Half of the men were past or current smokers (50.9%) while far fewer women smoked (16.6%), and these frequencies were lower among patients (47.2% and 3.3% among men and women, respectively). However, the quantity of cigarettes smoked was higher among smokers with Alzheimer's disease (median 15.0 versus 11.3 pack-years for men and 12.0 versus 6.0 pack-years for women). Weekly or daily consumption of alcohol was reported by 82.7% and 45.4% of men and women without Alzheimer's, respectively, and these proportions were lower among patients (66.7% and 35.0% among men and women, respectively). In Alzheimer's-free individuals 62.3% of the men and 47.3% of the women had a BMI >25 kg/m^2^, but the frequencies among cases were lower (41.6% and 41.8% among men and women, respectively).

**Table 1:** Characteristics of the sample population according to gender and to Alzheimer’s disease report at enrolment. AGRICAN Cohort, France. [IQR=interquartile range.]

		Men (N=61 191)										Women (N=48 106)								
		No Alzheimer’s disease					Alzheimer’s disease					No Alzheimer’s disease					Alzheimer’s disease			
		N=60 914					N=267					N=47 555					N=551			
		N	%	Median	IQR		N	%	Median	IQR		N	%	Median	IQR		N	%	Median	IQR
**Sociodemographic and lifestyle data**																				
Age				60	46–72				79	75–83				64	51–75				83	78–86
Education																				
	Primary	22 843	37.5				212	79.4				24 334	51.2				488	88.6		
	Secondary	30 070	49.4				49	18.4				18 073	38.0				59	10.7		
	Higher	8001	13.1				6	2.2				5148	10.8				4	0.7		
Tobacco smoking																				
	Never smoked	29 926	49.1				141	52.8				39 648	83.4				533	96.7		
	Current/past smoker	30 988	50.9				126	47.2				7907	16.6				18	3.3		
	Number pack/years			11.3	5–22				15.0	7–31				6.0	2–13				12.0	2–29
Body mass index																				
	<18.5	302	0.5				6	2.3				1219	2.6				37	6.7		
	18.5–25	22 441	36.8				108	40.4				23 801	50.1				284	51.5		
	25–30	29 190	47.9				111	41.6				15 859	33.3				163	29.6		
	>30	8981	14.8				42	15.7				6676	14.0				67	12.2		
	Value			26.0	24–28				25.8	24–29				24.7	22–28				24.0	21–27
Alcohol consumption																				
	Never	5008	8.2				56	21.0				14 115	29.7				266	48.3		
	Monthly	5510	9.1				33	12.4				11 853	24.9				92	16.7		
	Weekly	23 014	37.8				67	25.1				15 826	33.3				112	20.3		
	Daily	27 382	44.9				111	41.6				5761	12.1				81	14.7		
Exposure data *																				
	Work on a farm						512					42 161					512			
	No	7936	14.0				17	3.3				7487	17.7				17	3.3		
	Yes	48 533	86.0				195	96.7				34 674	82.3				195	96.7		
Pesticide poisoning							235					45 033					514			
	No	55 206	94.1				220	93.6				44 349	98.5				500	97.3		
	Yes	3440	5.9				15	6.4				684	1.5				14	2.7		
Pesticide use							195					34 093					404			
	No	18 253	35.2				75	38.5				29 656	87.0				329	81.4		
	Yes	33 540	64.8				120	61.5				4437	13.0				75	18.6		

* Individuals with no missing data on covariates: age, education level, tobacco smoking, alcohol consumption, and body mass index.

### Association between Alzheimer's disease and sociodemographic characteristics

As shown in table 1, among the 818 patients with Alzheimer's disease, 691 (89.6%) questionnaires were completed by – or with the help of – a next of kin (versus 17% for non-Alzheimer's individuals); while 80 (9.8%) reported that they had responded themselves, this information was missing for 47 individuals (5.7%). Age at diagnosis was reported to be ≤60 years old for only 51 individuals (6.2%). The estimated delay between diagnosis and enrolment was 11 years.

Age was associated with Alzheimer's disease among both men (+13% per year of age) and women (+14% per year of age). Adjusting for age, the risk of Alzheimer's disease decreased with the level of education (versus primary school level: OR 0.69, 95% CI 0.54–0.87 and OR 0.56, 95% CI 0.31–1.03 respectively for secondary and higher level among men; OR 0.65, 95% CI 0.52–0.81 and OR 0.59, 95% CI 0.28–1.25 among women). Considering the number of pack-years and adjusting for age, smoking tended to slightly increase the risks among both men (OR 1.00, 95% CI 1.00–1.01) and women (OR 1.01, 95% CI 0.99–1.03). Risks were decreased with alcohol according to the frequency of consumption (versus non-drinkers: monthly OR 0.70, 95% CI 0.59–0.83, weekly: OR 0.55, 95% CI 0.48–0.64, daily: OR 0.57, 95% CI 0.48–0.65) and also with the BMI as a continuous variable (OR 0.95, 95% CI 0.94–0.97).

### Association between Alzheimer's disease and farm work

In the analysis only adjusted for age, individuals who had worked on a farm had an increased risk of Alzheimer's disease compared to non-agricultural participants (OR 1.52, 95% CI 1.13–2.04 globally; OR 1.75, 95% CI 1.08–2.81 among men; and OR 1.33, 95% CI 0.91–1.95 among women).

Adjusting for all covariates, the risk of Alzheimer's disease among individuals who had worked on a farm was lower but still increased among men (OR 1.81, 95% CI 0.92–3.57) and among women (OR 1.58, 95% CI 0.94–2.66). The strength of associations was comparable in those who reported growing crops (OR 1.80, 95% CI 0.91–3.56 among men and OR 1.58, 95% CI 0.93–2.66 among women) and those raising animals (OR 1.74, 95% CI 0.87–3.45 among men and OR 1.57, 95% CI 0.93–2.65 among women), knowing that two thirds of farmers in AGRICAN had practiced both.

### Association between Alzheimer's disease and exposure to pesticides

In our population, 33 660 men (64.7%) and 4512 women (13.1%) reported the use of pesticides in any job (agriculture or other industry). The risk of Alzheimer's disease among participants reporting pesticide use was higher in both sexes, although slightly lower among men (OR 1.14, 95% CI 0.85–1.53 versus OR 1.42, 95% CI 1.10–1.85 among women). The risk of Alzheimer's disease among participants who had been poisoned by pesticides was OR 1.64, 95% CI 1.12–2.40, higher among women (OR 2.33, 95% CI 1.33–4.06) than men (OR 1.48, 95% CI 0.85–1.53).

### Association between Alzheimer's disease and exposure to crops and livestock

Among participants who have worked on a farm, the risks of Alzheimer's disease related to specific crops and livestock were generally close to unity (table 2). The highest risks were observed for pigs (OR 1.38, 95% CI 1.01–1.89) and rapeseed (OR 1.45, 95% CI 1.00–2.11)among men, and sunflower among women (OR 1.55, 95% CI 0.90–2.66).

**Table 2:** Risks of Alzheimer’s according to sex and each crop and livestock. All models adjusted for age, education level, tobacco smoking, alcohol consumption, body mass index. Reference group consisted of non-agricultural participants. [nE=number of cases exposed; OR=odds ratio; CI=confidence interval.]

		Men				Women		
		nE	OR	95% CI		nE	OR	95% CI
Pesticide use		120	1.14	0.85–1.53		75	1.42	1.10–1.85
Work in agriculture		237	1.81	0.92–3.57		495	1.58	0.94–2.86
Livestock								
	Cattle	171	0.99	0.67–1.46		380	1.19	0.88–1.60
	Sheep	36	1.06	0.73–1.55		78	1.08	0.83–1.40
	Pigs	91	1.38	1.01–1.89		236	1.18	0.94–1.48
	Horses	90	0.98	0.71–1.33		115	1.02	0.80–1.29
	Poultry	83	1.05	0.76–1.45		315	1.23	0.94–1.60
Open field crops								
	Wheat/barley	138	1.11	0.79–1.56		197	1.11	0.89–1.37
	Corn	80	0.90	0.67–1.22		94	1.27	0.99–1.62
	Sunflower	18	1.15	0.70–1.89		15	1.55	0.90–2.66
	Rapeseed	36	1.45	1.00–2.11		27	1.17	0.78–1.77
	Field peas	12	0.80	0.44–1.45		19	1.09	0.68–1.76
	Beets	70	1.10	0.80–1.50		120	1.02	0.81–1.29
	Meadows	168	1.16	0.78–1.73		292	1.04	0.83–1.31
	Potatoes	93	1.22	0.89–1.67		184	0.89	0.71–1.11
Other crops								
	Vineyards	102	1.00	0.75–1.34		196	1.14	0.93–1.41
	Fruit-growing	60	1.10	0.78–1.57		129	1.15	0.90–1.47
	Vegetables	23	1.10	0.70–1.72		56	1.02	0.76–1.38
	Tobacco	26	1.05	0.69–1.60		50	0.96	0.71–1.31
	Greenhouses	7	1.05	0.49–2.28		15	1.32	0.77–2.27

### Association between Alzheimer's disease and pesticide use on specific crops and livestock

**Figure 1:**
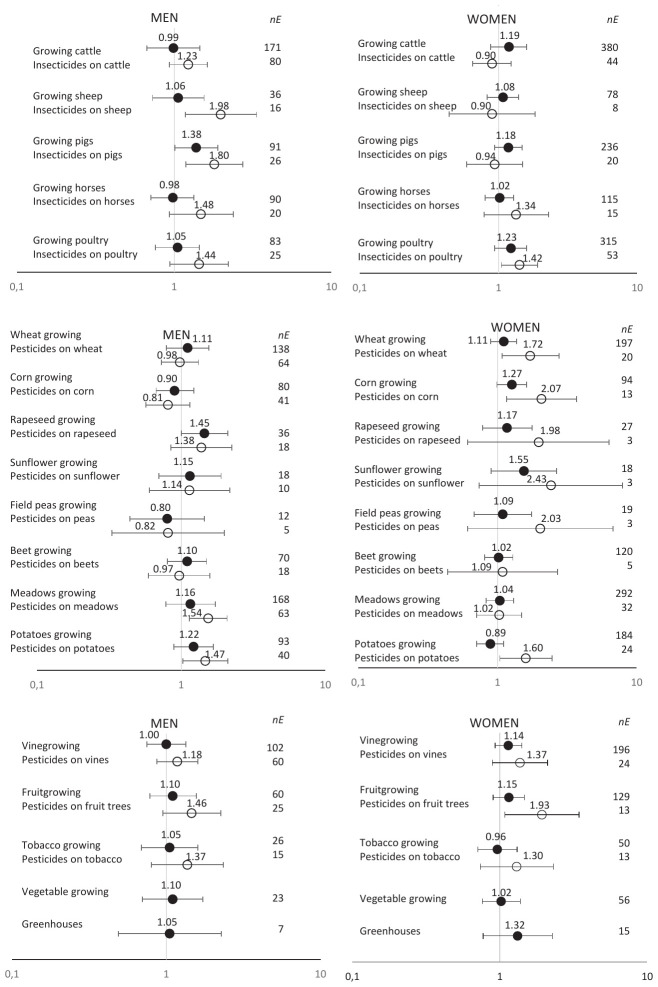
Risks of Alzheimer’s disease by gender according to the history of crops and livestock and the use of pesticides in farmers compared to non-farmers. AGRICAN cohort. Odds ratios (OR) and 95% confidence interval (CI) adjusted on age, education, alcohol consumption, smoking and body mass index. [nE=number of diseased individuals exposed.]

Among male farmers, risks were increased when pesticides were used on some crops: meadows (OR 1.54, 95% CI 1.14–2.08), potatoes (OR 1.47, 95% CI 1.03–2.10) fruit-growing (OR 1.46, 95% CI 0.95–2.25) (figure 1). Among women using pesticides, the increases in risks were more pronounced than among men for most crops, with OR approaching or >2, except for use on meadows, beets, tobacco or vineyards.

For pesticide use on animals, men had elevated risks, especially for sheep/goats (OR 1.98, 95% CI 1.18–3.34) and pigs (OR 1.80, 95% CI 1.19–2.74). Risks were also observed among women for pesticide use on horses (OR 1.34, 95% CI 0.79–2.29) and poultry (OR 1.42, 95% CI 1.05–1.91).

### Dose–effect relationships

Dose–effect relationships did not show clear patterns, but the analysis often involved small numbers. Only the surfaces of treated meadows were consistently associated with an increased risk in both men (+3% per 10 ha) and women (+ 18% every per 10 ha).

### Sensitivity analysis

Analysis using farmers growing other crops/livestock as the comparison group did not substantially change the results (table 4). Similarly, restricting the analysis to questionnaires completed by next of kin had little impact.

**Table 4:** Risks of Alzheimer’s disease in relation with pesticide use according to gender – sensitivity analysis. For specific crops and livestock, education level was considered in only two classes: primary school/secondary or high school. [OR=odds ratio; CI=confidence interval; *=numbers too limited].

		Main analysis				Comparison group (other farmers)				Questionnaire completed by next of kin				Alzheimer’s with an age at diagnosis reported>60 years				Main analysis adjusted on hypertension		
		NE	OR ^a^	95% CI ^a^		NE	OR ^a^	95% CI ^a^		NE	OR ^a^	95% CI ^a^		NE	OR ^a^	95% CI ^a^		NE	OR ^a^	95% CI ^a^
**Men**																				
Work on a farm		237	1.81	0.92–3.57			–	–		190	1.67	0.73–3.85		173	1.96	1.10–3.48		233	1.76	0.89–3.46
Use of pesticide in any job		120	1.14	0.85–1.53			–	–		97	1.28	0.91–1.79		92	1.31	0.93–1.86		113	1.12	0.82–1.52
Insecticides on livestock																				
	Cattle	80	1.23	0.93–1.63		80	1.19	0.89–1.58		64	1.23	0.89–1.68		57	1.28	0.91–1.78		78	1.22	0.92–1.63
	Sheep	16	1.98	1.18–3.34		16	1.93	1.15–3.25		14	2.00	1.13–3.55		13	2.25	1.26–4.01		16	2.03	1.21–3.42
	Pigs	26	1.80	1.19–2.74		26	1.77	1.16–2.69		23	1.52	0.96–2.40		17	1.58	0.95–2.65		25	1.77	1.15–2.71
	Horses	20	1.48	0.93–2.37		20	1.46	0.91–2.34		18	1.33	0.80–2.22		17	1.77	1.06–2.97		20	1.53	0.95–2.45
	Poultry	25	1.44	0.94–2.21		25	1.41	0.92–2.16		22	1.29	0.81–2.05		18	1.46	0.88–2.41		24	1.39	0.90–2.15
Pesticides on open field crops																				
	Wheat/barley	64	0.98	0.73–1.32		64	0.94	0.69–1.26		52	0.97	0.69–1.35		51	1.15	0.82–1.61		60	0.92	0.68–1.25
	Corn	41	0.81	0.57–1.15		41	0.77	0.54–1.09		32	0.82	0.55–1.22		29	0.85	0.57–1.29		39	0.79	0.55–1.12
	Rapeseed	18	1.38	0.85–2.25		18	1.32	0.81–2.16		14	1.48	0.84–2.61		14	1.60	0.92–2.81		18	1.42	0.87–2.32
	Sunflower	10	1.14	0.60–2.17		10	1.08	0.57–2.06		7	1.16	0.54–2.53		8	1.36	0.66–2.80		10	1.19	0.62–2.26
	Field peas	5	0.81	0.33–1.99		5	0.77	0.32–1.89		5	1.23	0.49–3.06		4	0.97	0.36–2.64		5	0.83	0.34–2.04
	Beets	18	0.97	0.59–1.58		18	0.94	0.58–1.53		18	1.04	0.63–1.72		16	1.24	0.73–2.09		17	0.93	0.57–1.54
	Meadows	63	1.54	1.14–2.08		63	1.49	1.10–2.01		53	1.49	1.07–2.07		45	1.57	1.10–2.23		60	1.48	1.09–2.01
	Potatoes	40	1.47	1.03–2.10		40	1.44	1.01–2.06		30	1.14	0.76–1.72		32	1.64	1.10–2.45		40	1.50	1.05–2.14
Pesticides on other crops																				
	Vineyards	60	1.18	0.87–1.60		60	1.15	0.85–1.56		48	1.06	0.75–1.49		45	1.20	0.84–1.70		57	1.14	0.83–1.55
	Fruit growing	25	1.46	0.95–2.25		25	1.43	0.93–2.19		18	1.28	0.76–2.13		19	1.50	0.92–2.46		13	1.43	0.92–2.21
	Tobacco	15	1.37	0.80–2.34		15	1.34	0.79–2.29		12	1.23	0.67–2.25		12	1.50	0.83–2.74		15	1.39	0.81–2.36
**Women**																				
Work on a farm		495	1.58	0.94–2.66			–	–		434	1.16	0.66–2.07		380	1.43	1.02–2.00		484	1.57	0.93–2.63
Use of pesticide in any job		75	1.42	1.10–1.85		75	1.41	1.08–1.84		68	1.37	1.04–1.81		54	1.27	0.94–1.72		71	1.42	1.09–1.85
Insecticides on livestock																				
	Cattle	44	0.90	0.65–1.23		44	0.89	0.64–1.22		41	0.96	0.69–1.34		34	0.92	0.64–1.32		44	0.93	0.67–1.28
	Sheep	8	0.90	0.44–1.84		8	0.89	0.43–1.83		7	0.84	0.39–1.83		4	0.57	0.21–1.55		8	0.95	0.46–1.96
	Pigs	20	0.94	0.59–1.49		20	0.93	0.58–1.48		18	0.80	0.49–1.31		17	1.04	0.63–1.72		20	0.99	0.62–1.57
	Horses	15	1.34	0.79–2.29		15	1.34	0.78–2.28		13	1.08	0.60–1.91		10	1.15	0.60–2.20		15	1.42	0.83–2.44
	Poultry	53	1.42	1.05–1.91		53	1.40	1.04–1.89		48	1.32	0.97–1.82		38	1.31	0.93–1.86		52	1.45	1.07–1.96
Pesticides on open field crops																				
	Wheat/barley	20	1.72	1.08–2.76		20	1.71	1.07–2.74		16	1.35	0.80–2.28		14	1.53	0.87–2.66		20	1.85	1.15–2.96
	Corn	13	2.07	1.16–3.69		13	2.06	1.16–3.67		11	1.99	1.05–3.76		11	2.34	1.25–4.39		13	2.15	1.20–3.83
	Rapeseed	3	1.98	0.61–6.35		3	1.96	0.61–6.30		2	*	*		2	*	*		3	2.03	0.63–6.55
	Sunflower	3	2.43	0.74–7.93		3	2.41	0.74–7.88		3	2.45	0.73–8.27		2	*	*		3	2.43	0.74–7.99
	Field peas	3	2.03	0.61–6.78		3	2.02	0.60–6.74		3	2.19	0.65–7.42		3	2.71	0.81–9.14		3	2.30	0.69–7.64
	Beets	5	1.09	0.44–2.70		5	1.08	0.42–2.67		4	0.85	0.31–2.35		3	0.85	0.27–2.70		5	1.17	0.47–2.91
	Meadows	32	1.03	0.71–1.49		32	1.02	0.70–1.48		28	0.93	0.63–1.39		24	0.98	0.64–1.51		32	1.05	0.72–1.53
	Potatoes	24	1.60	1.04–2.46		24	1.58	1.03–2.44		23	1.39	0.89–2.17		20	1.74	1.08–2.79		24	1.72	1.11–2.65
Pesticides on other crops																				
	Vineyards	24	1.37	0.89–2.11		24	1.36	0.88–2.09		20	1.13	0.71–1.81		16	1.16	0.69–1.95		24	1.45	0.94–2.23
	Fruit growing	13	1.93	1.08–3.45		13	1.90	1.07–3.41		8	1.47	0.70–3.09		8	1.52	0.73–3.14		13	2.10	1.17–3.77
	Tobacco	13	1.30	0.74–2.31		13	1.29	0.73–2.29		11	1.10	0.59–2.06		7	0.91	0.42–1.95		12	1.30	0.72–2.36

^a^ Adjusted for age, education, alcohol consumption, smoking and body mass index. Adjustment for smoking among women was not possible for rapeseed and fruit-growing. The reference group consisted of non-agricultural participants (except for comparisons within farmers).

When considering only the men who reported an age at diagnosis of >60 years, risks increased for all crops and livestock in (up to 28% for beets), except for pigs. Among women, the trends were less consistent and in the two directions with reduced precision for some crops due to limited numbers.

Analysis with further adjustment on cardiovascular conditions (hypertension – see table 3; cardiac arrhythmia – results not shown) and diabetes (results not shown) barely affected the results.

**Table 3:** Risks of Alzheimer’s according to sex and quantitative metrics of pesticide use: duration and number of treated animals for livestock, duration and treated surface for crops. All models adjusted for age, education level, tobacco smoking, alcohol consumption, body mass index. No information was collected on pesticide use in greenhouses and on vegetables; no details were available regarding the duration or surface area of treatments on potatoes. The reference group consisted of non-agricultural participants. [nE=number of cases exposed; *: numbers too limited (nE<3); OR=odds ratio; CI=confidence interval.]

		Men				Men				Women				Women		
		Duration of pesticide use on animals or crops				# of animals or surfaces treated				Duration of pesticide use on animals or crops				# of animals or surfaces treated		
		nE	OR ^a^	95% CI		nE	OR ^a^	95% CI		nE	OR ^a^	95% CI		nE	OR ^a^	95% CI
Insecticides on livestock*																
	Cattle	33	0.89	0.68–1.15		19	0.96	0.84–1.10		21	0.94	0.68–1.30		17	1.00	0.96–1.04
	Sheep/goats	7	1.27	0.70–2.29		5	0.93	0.80–1.09		5	1.65	0.78–3.48		3	0.49	0.09–2.76
	Pigs	15	0.71	0.47–1.05		9	1.00	0.47–1.05		9	1.04	0.63–1.72		5	0.09	0.002–5.03
	Horses	13	0.66	0.38–1.15		7	0.80	0.11–5.66		7	1.13	0.58–2.20		5	1.33	0.18–9.86
	Poultry	15	1.04	0.99–1.00		10	1.00	0.99–1.00		19	1.16	0.85–1.59		19	0.99	0.95–1.03
Pesticides on open field crops																
	Wheat/barley	24	1.04	0.75–1.44		18	1.02	0.96–1.08		12	1.11	0.66–1.87		3	1.98	0.70–5.57
	Corn	18	1.06	0.70–1.59		12	0.99	0.83–1.19		6	1.17	0.55–2.51		3	3.35	0.70–16.0
	Rapeseed	6	0.93	0.50–1.74		2	*	*		1	*	*		1	*	*
	Sunflower	3	1.65	0.63–4.30		2	*	*		3	*	*		1	*	*
	Field peas	2	*	*		2	*	*		2	*	*		0	*	*
	Beets	10	0.66	0.40–1.10		5	1.04	0.96–1.14		5	1.28	0.45–3.62		2	*	*
	Meadows	44	1.03	0.82–1.28		28	1.03	1.01–1.06		22	1.02	0.75–1.37		16	1.18	1.04–1.32
Pesticides on other crops																
	Vineyards	24	1.12	0.84–1.47		16	0.92	0.69–1.22		12	0.95	0.63–1.44		8	0.92	0.58–1.46
	Fruit-growing	10	0.75	0.48–1.18		3	0.89	0.31–2.50		2	*	*		2	*	*
	Tobacco	5	1.03	0.53–2.01		2	*	*		6	1.31	0.60–2.88		2	*	*

^a^ OR are calculated for the increment of 10 years of pesticide use (for duration) or for the increment of 10 animals/10 hectares treated.

Transforming the age variable (logarithmic, quadratic, cubic splines) did not change the risk estimates.

## Discussion

In a large cohort in agriculture and related industries, we showed that farming and use of pesticides were associated with a higher risk of developing Alzheimer's disease, with variations according to the gender and the type of crops/livestock. Among men, elevated risks were observed for those growing rapeseed and rearing pigs, with more pronounced effect of insecticide use on pigs and sheep. Meadows and potatoes were also associated with an increased risk. Among women, increases were seen for the use of insecticides on poultry as well as most crops, but notably wheat/barley, corn, potatoes and fruit-growing plants.

The AGRICAN cohort offered the opportunity to analyze detailed farming exposures, considering gender. Women have rarely been included in agricultural studies, making our results even more original and relevant particularly as Alzheimer's disease affects women more.

An important strength of our study lies in the opportunity to reconstitute the agricultural exposome going as far as the type of crops/livestock and the information for pesticide use on each of them across the span of an individual's life, including the duration, number of animals and surfaces. Such a perspective is very important when studying a chronic disease like Alzheimer's: details enable to explore several exposure metrics, from general ones to more targeted (eg, use of pesticides on specific crops/animals). As >90% of diagnoses were made after age 65, retirement age for most people, we are confident that exposure almost always preceded the disease. Although some farmers continue working beyond retirement age, this is unlikely to substantially affect the temporal relationship between exposure and disease onset.

A limitation of this study is the reliance on retrospectively collected self-reported pesticide use, which may introduce differential recall bias. If such a bias were present, we would expect it to affect risk estimates across all exposures and equally on men and women, which was not the case. Moreover, prior work in the AGRICAN cohort has demonstrated substantial reliability for participants' reporting of occupational history and tasks performed (23). Future studies could further refine exposure assessment using the PESTIMAT crop-exposure matrix, combining detailed task histories with crop information to evaluate specific pesticide molecules and reduce exposure misclassification (24).

We acknowledge that the diagnosis of Alzheimer's disease, even though individuals were asked whether a doctor had diagnosed them, is a limitation. This data was prone to memory bias due to potential cognitive impairment. However, Alzheimer's disease must be seen as a continuum from normal cognition to mild, moderate and then severe stages (25). The early stages of the disease, confirmed by amyloid profile, do not necessarily preclude reliable answers to questionnaires, especially on past events. The speed of cognitive impairment is variable between individuals. In France, it has been estimated that the early stages would correspond to one third of the diagnosed patients (26). Moreover, AGRICAN participants could be assisted by a next-of-kin who completed the questionnaire for or with them (in about 90% of cases). Surrogate respondents may not know all about the life of the participants, but our results remained consistent when we restricted the analysis to questionnaires completed by a next of kin. Moreover, the proportion of missing data was generally close when the questionnaire was answered by the participant himself or a next of kin. These elements are not in favor of an important bias related to questionnaires completed by a next of kin. We may have missed cases present in the cohort but undiagnosed. Indeed, as demonstrated in other studies, significant barriers to healthcare have been described for agricultural populations, showing that they suffer from geographic isolation, a shortage of specialized healthcare professionals, financial difficulties, and reluctance to ask for help, which leads to delayed diagnoses and lower access to treatment, especially for aging populations (27). To address potential bias related to this issue as the reference group was outside agriculture, we also performed a sensitivity analysis with farmers as the reference group, that showed little impact on the results. In any case, we cannot completely rule out that classification errors about the disease may have occurred.

In our analysis, we considered the main risk factors that have been associated with Alzheimer's disease: age, gender, educational level, tobacco smoking, alcohol consumption and BMI. We ran separate analyses on men and women, finding that associations with exposure differed according to gender. We can hypothesize that sensitivity to agricultural exposures varies according to gender; certain exposures differ between men and women by their frequency, intensity or type of pesticides; or the combination with other risk factors plays a different role (depression, education, hormones, social environment, etc), but we cannot completely rule out that these differences are due to chance. We could not consider APOE4, a well-established genetic risk factor, as biological samples were not available (9). We ran further models adjusted on some self-reported cardiovascular factors: the power of our analysis decreased, but our results were globally unchanged.

Studies exploring the links between Alzheimer's disease and farming/ pesticides are scarce. Some of them have relied on rough data on the diagnosis from death certificates (28–30) or limited definition of exposure such as a single job title (28, 29) or a single question on pesticide use (31, 32). Most had a very low number of exposed cases (31–34) or insufficient consideration of major confounders (28, 29, 31). Despite these limitations, some of them have found positive results. In the US, an analysis based on death certificates and job titles found an increase of Alzheimer's disease in farmers, especially among those who owned farms (OR 1.76, 95% CI 1.04–2.81). A Canadian study found a doubling in risk in a case–control study in people aged ≥65 years from 36 cities (258 cases and 535 controls) based on a precise clinical diagnosis but a single question on pesticide use (OR 2.17, 95% CI 1.25–3.79) (34). A comparable increase, but more imprecise, was found in an Australian hospital case–control study (170 cases and 170 controls) with an OR of 2.54 (95% CI 0.41–27.06) for individuals with occupational use of organophosphates (33). Two more recent case–control studies based their exposure assessment on serum organochlorine levels, one in Texas (86 cases and 79 controls) found an association for the highest DDE levels OR 4.18, 95% CI 2.54–5.82) (35), while the other one observed an increase in some organochlorine levels in patients in Delhi (70 cases and 75 controls) but without risk estimates (36).

Only four prospective cohorts have investigated the association between pesticides and Alzheimer's disease, but all have found positive associations, with the risk more than doubling in three of them. All were based on clinical diagnosis, but the number of patients was limited (N=36–399). Two have used detailed information on jobs with details of pesticide exposure (37, 38) while the two others have questioned the link with agriculture (39) or pesticide groups (38, 40). Based on 36 cases, the Canadian study (38) found a doubling in risk for farmers [risk ratio (RR) 2.59, 95% CI 1.05–5.6] and observed a quadrupling in the risk for people reporting the use of fumigants or defoliants (RR 4.35, 95% CI 1.05–17.90). Ten years later, the same cohort was analyzed for the association between the disease (N=399) and the plasmatic levels of organochlorines, but no clear associations were found (40, 41). In France, a cohort of 1507 elderly people, identifying 96 incident cases, combined job histories with a job-exposure matrix and found that male farmers were twice as much at risk of developing Alzheimer's disease (OR 2.4, 95% CI 1.0–5.6) while no increase in risk was identified among women (37). In Utah (USA), within an agricultural community (N=3084) where 344 patients were diagnosed with Alzheimer's disease, an increased risk was observed globally in those using pesticides (RR 1.42, 95% CI 1.06–1.91) and for use of organophosphates or organochlorines separately (38).

Our results are consistent with other studies but not easily comparable as none of them explored specific crops or livestock. Considering the three cohorts with positive results, they covered a diversity of contexts. In Canada, most participants were living in urban settings and, for those in rural places, the most probable crops were forests, cereals and canola (42). In Utah, the most prevalent crops were alfalfa and cereal (43). In France, the main crop was grapes. None of these studies explored the link with livestock, although raising livestock is an important industry in Utah (cattle and sheep), Canada (cattle, pigs and poultry) and in France (see AGRICAN data). Uses on animals are frequently omitted when assessing pesticide exposures because of a biased representation of these products, according to which they are good for animal health. However, in our study, associations were found with insecticide use on animals. This result is in line with those observed among British sheep farmers, who had handled or were poisoned by pesticides and had an increased risk of dementia (OR 6.94, 95% CI 3.44–14.0) (44).

The link with pesticide use on animals would tend to point towards the group of insecticides, but due to associations with a number of crops, we cannot exclude the role of other molecules such as herbicides or fungicides.

The present results should also be compared with those studies that have focused on chronic cognitive disorders identified in farmers for repeated pesticide exposures. More than 50 studies have showed a consistent association between pesticide exposure (especially organophosphates) and cognitive disorders (45–49). As cognitive disorders are frequently the first event in Alzheimer's disease, these results raise the concern that the impact of pesticides on cognitive function could be predictive for the development of Alzheimer's disease (50). Toxicological data also support our results, showing that several mechanisms could explain the occurrence of the disease such as neuronal degeneration, oxidative stress and mitochondrial dysfunction of role in amyloid beta accumulation (51–53).

### Concluding remarks

Few studies have been conducted on the role of pesticides in the occurrence of Alzheimer's disease, but it is specific interest for several reasons: (i) Alzheimer's disease is a major public health concern in our aging societies, and we have very little information on its environmental etiology; (ii) even if data are scarce, the existing studies have found consistent positive associations as far as they have been conducted with accurate information on the disease, exposure assessment and covariates; (iii) several studies have shown associations between cognitive impairment and pesticide exposure and these outcomes are predictive of Alzheimer's disease. This study brings new results and demonstrates that refining exposure parameters is of interest, including examining the context in which pesticides are used and considering men and women separately.

## Supplementary material

Supplementary materials

## References

[1] Nichols E, Steinmetz JD, Vollset SE, Fukutaki K, Chalek J, Abd-Allah F et al.; GBD 2019 Dementia Forecasting Collaborators. Estimation of the global prevalence of dementia in 2019 and forecasted prevalence in 2050: an analysis for the Global Burden of Disease Study 2019. Lancet Public Health 2022 Feb;7(2):e105–25. 10.1016/S2468-2667(21)00249-8 34998485 PMC8810394

[2] Dementia [Internet]. [cited 21 August 2024]. Available from: https://www.who.int/news-room/fact-sheets/detail/dementia

[3] International AD. World Alzheimer Report 2023: Reducing Dementia Risk: Never too early, never too late. 21 Sept 2023 [cited 21 August 2024]; Available from: https://www.alzint.org/resource/world-alzheimer-report-2023/

[4] Gustavsson A, Norton N, Fast T, Frölich L, Georges J, Holzapfel D et al. Global estimates on the number of persons across the Alzheimer's disease continuum. Alzheimers Dement [Internet]. [cited 6 February 2023]. Available from: https://onlinelibrary.wiley.com/doi/abs/10.1002/alz.12694 10.1002/alz.12694 35652476

[5] Bakhta K, Cecillon E, Lacombe E, Lamy M, Leboucher A, Philippe J. Alzheimer’s disease and neurodegenerative diseases in France. Lancet 2019 Aug;394(10197):466–7. 10.1016/S0140-6736(19)31633-2 31402022

[6] Mukadam N, Wolters FJ, Walsh S, Wallace L, Brayne C, Matthews FE et al. Changes in prevalence and incidence of dementia and risk factors for dementia: an analysis from cohort studies. Lancet Public Health 2024 Jul;9(7):e443–60. 10.1016/S2468-2667(24)00120-8 38942556

[7] Vieira RT, Caixeta L, Machado S, Silva AC, Nardi AE, Arias-Carrión O et al. Epidemiology of early-onset dementia: a review of the literature. Clin Pract Epidemiol Ment Health 2013 Jun;9:88–95. 10.2174/1745017901309010088 23878613 PMC3715758

[8] Krüger J, Aaltonen M, Aho K, Heikkinen S, Kivisild A, Lehtonen A et al. Incidence and Prevalence of Early-Onset Dementia in Finland. Neurology 2024 Aug;103(4):e209654. 10.1212/WNL.0000000000209654 39047214 PMC11314947

[9] Tsuboi Y, Josephs KA, Cookson N, Dickson DW. APOE E4 is a determinant for Alzheimer type pathology in progressive supranuclear palsy. Neurology 2003 Jan;60(2):240–5. 10.1212/01.WNL.0000044340.37138.A9 12552038

[10] Livingston G, Huntley J, Liu KY, Costafreda SG, Selbæk G, Alladi S et al. Dementia prevention, intervention, and care: 2024 report of the *Lancet* standing Commission. Lancet 2024 Aug;404(10452):572–628. 10.1016/S0140-6736(24)01296-0 39096926

[11] He SY, Su WM, Wen XJ, Lu SJ, Cao B, Yan B et al. Non-Genetic Risk Factors of Alzheimer’s Disease: An Updated Umbrella Review. J Prev Alzheimers Dis 2024;11(4):917–27. 10.14283/jpad.2024.100 39044503 PMC11266231

[12] Santibáñez M, Bolumar F, García AM. Occupational risk factors in Alzheimer’s disease: a review assessing the quality of published epidemiological studies. Occup Environ Med 2007 Nov;64(11):723–32. 10.1136/oem.2006.028209 17525096 PMC2078415

[13] Gunnarsson LG, Bodin L. Occupational Exposures and Neurodegenerative Diseases-A Systematic Literature Review and Meta-Analyses. Int J Environ Res Public Health 2019 Jan;16(3):E337. 10.3390/ijerph16030337 30691095 PMC6388365

[14] Yan D, Zhang Y, Liu L, Yan H. Pesticide exposure and risk of Alzheimer’s disease: a systematic review and meta-analysis. Sci Rep 2016 Sep;6:32222. 10.1038/srep32222 27581992 PMC5007474

[15] FAOSTAT [Internet]. [cited 28 September 2022]. Avalable from: https://www.fao.org/faostat/en/#data/RP/visualize

[16] Levêque-Morlais N, Tual S, Clin B, Adjemian A, Baldi I, Lebailly P. The AGRIculture and CANcer (AGRICAN) cohort study: enrollment and causes of death for the 2005-2009 period. Int Arch Occup Environ Health 2015 Jan;88(1):61–73. 10.1007/s00420-014-0933-x 24599726

[17] Alzheimer’s Association. 2019 Alzheimer’s disease facts and figures. Alzheimers Dement 2019;15(3):321–87. 10.1016/j.jalz.2019.01.010

[18] Daviglus ML, Bell CC, Berrettini W, Bowen PE, Connolly ES Jr, Cox NJ et al. NIH state-of-the-science conference statement: preventing Alzheimer’s disease and cognitive decline. NIH Consens State Sci Statements 2010 Apr;27(4):1–30. 20445638

[19] Carter CL, Resnick EM, Mallampalli M, Kalbarczyk A. Sex and gender differences in Alzheimer’s disease: recommendations for future research. J Womens Health (Larchmt) 2012 Oct;21(10):1018–23. 10.1089/jwh.2012.3789 22917473

[20] Hebert LE, Weuve J, Scherr PA, Evans DA. Alzheimer disease in the United States (2010-2050) estimated using the 2010 census. Neurology 2013 May;80(19):1778–83. 10.1212/WNL.0b013e31828726f5 23390181 PMC3719424

[21] Mielke MM, Vemuri P, Rocca WA. Clinical epidemiology of Alzheimer’s disease: assessing sex and gender differences. Clin Epidemiol 2014 Jan;6:37–48. 10.2147/CLEP.S37929 24470773 PMC3891487

[22] Nebel RA, Aggarwal NT, Barnes LL, Gallagher A, Goldstein JM, Kantarci K et al. Understanding the impact of sex and gender in Alzheimer’s disease: A call to action. Alzheimers Dement 2018 Sep;14(9):1171–83. 10.1016/j.jalz.2018.04.008 29907423 PMC6400070

[23] Tual S, Lemarchand C, Giovannini J, Boulanger M, Meryet-Figuiere M, Talibov M et al. Reliability of baseline self-reported information in the AGRICAN cohort. Cancer Causes Control 2022 Feb;33(2):331–42. 10.1007/s10552-021-01516-z 34984593

[24] Baldi I, Carles C, Blanc-Lapierre A, Fabbro-Peray P, Druet-Cabanac M, Boutet-Robinet E et al.; PESTIMAT Group. A French crop-exposure matrix for use in epidemiological studies on pesticides: PESTIMAT. J Expo Sci Environ Epidemiol 2017 Jan;27(1):56–63. 10.1038/jes.2015.72 26696463

[25] Davis M, O Connell T, Johnson S, Cline S, Merikle E, Martenyi F et al. Estimating Alzheimer’s Disease Progression Rates from Normal Cognition Through Mild Cognitive Impairment and Stages of Dementia. Curr Alzheimer Res 2018;15(8):777–88. 10.2174/1567205015666180119092427 29357799 PMC6156780

[26] Gabelle A, Guéry M, Doutriaux A, Bettayeb K. Forecasting the Prevalence of Alzheimer’s Disease at Mild Cognitive Impairment and Mild Dementia Stages in France in 2022. J Prev Alzheimers Dis 2023;10(2):259–66. 10.14283/jpad.2023.22 36946453

[27] Pérès K, Brayne C, Matharan F, Grasset L, Helmer C, Letenneur L et al. Trends in Prevalence of Dementia in French Farmers from Two Epidemiological Cohorts. J Am Geriatr Soc 2017 Feb;65(2):415–20. 10.1111/jgs.14575 27991652

[28] Park RM, Schulte PA, Bowman JD, Walker JT, Bondy SC, Yost MG et al. Potential occupational risks for neurodegenerative diseases. Am J Ind Med 2005 Jul;48(1):63–77. 10.1002/ajim.20178 15940722

[29] Schulte PA, Burnett CA, Boeniger MF, Johnson J. Neurodegenerative diseases: occupational occurrence and potential risk factors, 1982 through 1991. Am J Public Health 1996 Sep;86(9):1281–8. 10.2105/AJPH.86.9.1281 8806381 PMC1380592

[30] Koeman T, Schouten LJ, van den Brandt PA, Slottje P, Huss A, Peters S et al. Occupational exposures and risk of dementia-related mortality in the prospective Netherlands Cohort Study. Am J Ind Med 2015 Jun;58(6):625–35. 10.1002/ajim.22462 25943788

[31] French LR, Schuman LM, Mortimer JA, Hutton JT, Boatman RA, Christians B. A case-control study of dementia of the Alzheimer type. Am J Epidemiol 1985 Mar;121(3):414–21. 10.1093/oxfordjournals.aje.a114013 4014131

[32] Gauthier E, Fortier I, Courchesne F, Pepin P, Mortimer J, Gauvreau D. Environmental pesticide exposure as a risk factor for Alzheimer’s disease: a case-control study. Environ Res 2001 May;86(1):37–45. 10.1006/enrs.2001.4254 11386739

[33] Gun RT, Korten AE, Jorm AF, Henderson AS, Broe GA, Creasey H et al. Occupational risk factors for Alzheimer disease: a case-control study. Alzheimer Dis Assoc Disord 1997 Mar;11(1):21–7. 10.1097/00002093-199703000-00005 9071441

[34] The Canadian Study of Health and Aging. The Canadian Study of Health and Aging: risk factors for Alzheimer’s disease in Canada. Neurology 1994 Nov;44(11):2073–80. 10.1212/WNL.44.11.2073 7969962

[35] Richardson JR, Roy A, Shalat SL, von Stein RT, Hossain MM, Buckley B et al. Elevated serum pesticide levels and risk for Alzheimer disease. JAMA Neurol 2014 Mar;71(3):284–90. 10.1001/jamaneurol.2013.6030 24473795 PMC4132934

[36] Singh N, Chhillar N, Banerjee B, Bala K, Basu M, Mustafa M. Organochlorine pesticide levels and risk of Alzheimer’s disease in north Indian population. Hum Exp Toxicol 2013 Jan;32(1):24–30. 10.1177/0960327112456315 22899726

[37] Baldi I, Lebailly P, Mohammed-Brahim B, Letenneur L, Dartigues JF, Brochard P. Neurodegenerative diseases and exposure to pesticides in the elderly. Am J Epidemiol 2003 Mar;157(5):409–14. 10.1093/aje/kwf216 12615605

[38] Hayden KM, Norton MC, Darcey D, Ostbye T, Zandi PP, Breitner JC et al.; Cache County Study Investigators. Occupational exposure to pesticides increases the risk of incident AD: the Cache County study. Neurology 2010 May;74(19):1524–30. 10.1212/WNL.0b013e3181dd4423 20458069 PMC2875926

[39] Tyas SL, Manfreda J, Strain LA, Montgomery PR. Risk factors for Alzheimer’s disease: a population-based, longitudinal study in Manitoba, Canada. Int J Epidemiol 2001 Jun;30(3):590–7. 10.1093/ije/30.3.590 11416089

[40] Medehouenou TC, Ayotte P, Carmichael PH, Kröger E, Verreault R, Lindsay J et al. Plasma polychlorinated biphenyl and organochlorine pesticide concentrations in dementia: the Canadian Study of Health and Aging. Environ Int 2014 Aug;69:141–7. 10.1016/j.envint.2014.04.016 24846810

[41] Medehouenou TC, Ayotte P, Carmichael PH, Kröger E, Verreault R, Lindsay J et al. Exposure to polychlorinated biphenyls and organochlorine pesticides and risk of dementia, Alzheimer’s disease and cognitive decline in an older population: a prospective analysis from the Canadian Study of Health and Aging. Environ Health 2019 Jun;18(1):57. 10.1186/s12940-019-0494-2 31200706 PMC6570931

[42] Recensement de l'agriculture [Internet] [Agricultural census]. [cited 21 August 2024]. Available from: https://www.statcan.gc.ca/fr/recensement-agriculture

[43] Hilton J, Gentillon J. United States Department of Agriculture National Agricultural Statistics Service Mountain Region, Utah Field Office. Available from https://www.nass.usda.gov/Statistics_by_State/Utah/Publications/Annual_Statistical_Bulletin/2023-Agricultural-Statistics.pdf

[44] Povey AC, McNamee R, Alhamwi H, Stocks SJ, Watkins G, Burns A et al. Pesticide exposure and screen-positive neuropsychiatric disease in British sheep farmers. Environ Res 2014 Nov;135:262–70. 10.1016/j.envres.2014.09.008 25462674

[45] Bayrami M, Hashemi T, Malekirad AA, Ashayeri H, Faraji F, Abdollahi M. Electroencephalogram, cognitive state, psychological disorders, clinical symptom, and oxidative stress in horticulture farmers exposed to organophosphate pesticides. Toxicol Ind Health 2012 Feb;28(1):90–6. 10.1177/0748233711407243 21632574

[46] Muñoz-Quezada MT, Lucero BA, Iglesias VP, Muñoz MP, Cornejo CA, Achu E et al. Chronic exposure to organophosphate (OP) pesticides and neuropsychological functioning in farm workers: a review. Int J Occup Environ Health 2016 Jan;22(1):68–79. 10.1080/10773525.2015.1123848 27128815 PMC4894272

[47] Dobbins DL, Chen H, Cepeda MJ, Berenson L, Talton JW, Anderson KA et al. Comparing impact of pesticide exposure on cognitive abilities of Latinx children from rural farmworker and urban non-farmworker families in North Carolina. Neurotoxicol Teratol 2022;92:107106. 10.1016/j.ntt.2022.107106 35654325 PMC9361037

[48] Mora AM, Baker JM, Hyland C, Rodríguez-Zamora MG, Rojas-Valverde D, Winkler MS et al. Pesticide exposure and cortical brain activation among farmworkers in Costa Rica. Neurotoxicology 2022 Dec;93:200–10. 10.1016/j.neuro.2022.10.004 36228750 PMC10014323

[49] Hernández AF, González-Alzaga B, López-Flores I, Lacasaña M. Systematic reviews on neurodevelopmental and neurodegenerative disorders linked to pesticide exposure: methodological features and impact on risk assessment. Environ Int 2016;92-93:657–79. 10.1016/j.envint.2016.01.020 26896854

[50] Expertise collective, éditeur. Effects of pesticides on health – New data [Internet]. INSERM, EDP Sciences; 2022 [cited 6 Feburary 2023]. Available from: https://laboutique.edpsciences.fr/produit/1257/9782759827794/effects-of-pesticides-on-health

[51] Dhapola R, Sharma P, Kumari S, Bhatti JS, HariKrishnaReddy D. Environmental Toxins and Alzheimer’s Disease: a Comprehensive Analysis of Pathogenic Mechanisms and Therapeutic Modulation. Mol Neurobiol 2024 Jun;61(6):3657–77. 10.1007/s12035-023-03805-x 38006469

[52] Tang BL. Neuropathological Mechanisms Associated with Pesticides in Alzheimer’s Disease. Toxics 2020 Mar;8(2):21. 10.3390/toxics8020021 32218337 PMC7355712

[53] Mostafalou S, Abdollahi M. Pesticides and human chronic diseases: evidences, mechanisms, and perspectives. Toxicol Appl Pharmacol 2013 Apr;268(2):157–77. 10.1016/j.taap.2013.01.025 23402800

