# Supplementary material for: Farming exposures and Alzheimer's disease: cross-sectional analysis within the French AGRICAN cohort
Source: Scand J Work Environ Health. 2026 Apr 30;52(3):310–21. doi: 10.5271/sjweh.4284 (PMC13185533; doi:10.5271/sjweh.4284)
Supplement: Supplementary materials [file SJWEH-52-310-S001.pdf]

Farming exposures and Alzheimer's disease: cross-sectional analysis within the French AGRICAN cohort[1]

by Victoire Madeline, MD, Angéline Galvin, PhD, Lucie De Graaf, PhD, Julien Engelhardt, PhD, Pierre Lebailly PhD, Isabelle Baldi, PhD

1. Supplementary material
2. Correspondence to: Isabelle Baldi, Equipe EPICENE, INSERM U 1219, Bordeaux Population Health Centre, 146 rue Léo Saignat, 33076 Bordeaux, France. [E-mail: Isabelle.Baldi@u-bordeaux.fr]

S1: Risks of Alzheimer's according to sex for various exposure metrics

	Men			Women		
	nE	*OR*	*95% CI*	*nE*	*OR*	*95% CI*
**Pesticide Use**	*120*	*1.14*	*0.85-1.53*	**75**	**1.42**	**1.10-1.85**
**Work in agriculture**	*237*	*1.81*	*0.92-3.57*	495	1.58	0.94-2.86
**Livestock**						
Cattle	*186*	1.77	0.88-3.53	*410*	1.60	0.94-2.73
Sheep/goats	*41*	1.47	0.75-2.90	*93*	**1.83**	**1.01-3.29**
Pigs	*91*	1.44	0.79-2.64	*256*	**1.98**	**1.14-3.46**
Horses	*95*	1.30	0.71-2.38	*130*	**1.84**	**1.03-3.29**
Poultry	*88*	1.29	0.70-2.37	*340*	**1.80**	**1.05-3.07**
**Open field crops**						
Wheat/barley	*138*	1.16	0.65-2.06	*222*	**1.97**	**1.13-3.44**
Corn	*85*	1.26	0.69-2.29	*104*	**2.08**	**1.15-3.76**
Rapeseed	*41*	1.87	0.98-3.55	*27*	1.97	0.97-4.02
Sunflower	*18*	1.82	0.83-3.98	*15*	**2.55**	**1.16-5.61**
Field peas	*12*	1.34	0.57-3.16	*24*	1.97	0.98-3.96
Beets	*70*	1.42	0.76-2.65	*135*	1.71	0.95-3.03
Meadows	*188*	1.37	0.78-2.42	*307*	**1.75**	**1.02-3.00**
Potatoes	*103*	1.41	0.77-2.56	*209*	1.66	0.95-2.91
**Other crops**						
Vineyards	*107*	1.48	0.82-2.66	*211*	**1.81**	**1.06-3.12**
Fruit-growing	*60*	1.30	0.69-2.44	*139*	**1.85**	**1.05-3.25**
Tobacco	*26*	1.48	0.71-3.10	*55*	1.52	0.82-2.80
Vegetables	*28*	1.41	0.71-2.83	*76*	**1.99**	**1.09-3.62**
Greenhouses	*7*	1.65	0.61-4.45	*15*	2.08	0.97-4.46

nE: number of cases exposed; All models adjusted for Age, Education Level, Tobacco Smoking, Alcohol Consumption, BMI. Reference group consisted of non-agricultural participants.

S2: Risks of Alzheimer's disease according to sex and quantitative metrics for livestock (duration, maximal number of animals) and crops (duration and surface)

	**Men** *Duration of crop growing (per 10 yr) or duration of animal raising (per 10 yr)*			**Men** *# of animals (per ten) or crop surfaces (per 10 ha)*			**Women** Duration of crop growing (per 10 yr) or duration of animal raising (per 10 yr)			**Women** *# of animals (per ten) or crop surfaces (per 10 ha)*		
	nE	***OR***	***95% CI***	***nE***	***OR***	***95% CI***	***nE***	***OR***	***95% CI***	***nE***	***OR***	***95% CI***
**LIVESTOCK***												
Cattle	*107*	1.02	0.90-1.15	*114*	1.00	0.99-1.01	*252*	1.01	0.93-1.11	*243*	1.00	0.99-1.01
Sheep/Goats	*23*	1.27	0.98-1.64	*24*	1.00	0.99-1.01	*38*	1.12	0.91-1.38	*47*	0.90	0.80-1.00
Pigs	*51*	**1.24**	**1.02-1.50**	*51*	1.00	0.99-1.01	*122*	1.02	0.89-1.16	*130*	0.70	0.21-2.41
Horses	*49*	1.23	0.99-1.51	*51*	0.99	0.85-1.17	*51*	0.81	0.64-1.03	*60*	1.00	0.99-1.01
Poultry	*34*	1.11	0.92-1.35	*36*	1.00	0.99-1.00	*157*	0.97	0.88-1.07	*138*	1.00	0.99-1.00
**OPEN FIELD CROPS**												
Wheat/barley	*138*	1.13	0.96-1.33	*44*	0.98	0.89-1.08	*197*	1.03	0.89-1.19	*57*	1.05	0.96-1.14
Corn	*80*	1.02	0.81-1.27	*27*	0.96	0.82-1.13	*94*	0.83	0.65-1.05	*22*	1.05	0.77-1.44
Sunflower	*18*	1.13	0.63-2.03	*5*	0.93	0.45-1.94	*15*	1.43	0.80-2.56	*6*	1.31	0.51-3.42
Rapeseed	*36*	0.92	0.61-1.40	*8*	0.94	0.57-1.55	*27*	1.32	0.88-1.99	*10*	1.23	0.60-2.51
Field peas	*12*	0.70	0.19-2.58	*0*	*	*	*19*	0.75	0.32-1.73	*0*	*	*
Beets	*70*	0.97	0.76-1.23	*19*	0.99	0.82-1.18	*120*	0.98	0.80-1.21	*37*	0.44	0.12-1.72
Meadows	*168*	1.07	0.94-1.22	*70*	1.00	0.99-1.06	*292*	0.94	0.84-1.05	*128*	1.02	0.99-1.06
Potatoes	*47*	1.00	0.85-1.18	*39*	1.00	0.94-1.07	*100*	1.06	0.93-1.20	*84*	1.00	0.97-1.04
**OTHER CROPS**												
Vineyards	*102*	1.04	0.88-1.24	*30*	0.98	0.85-1.13	*196*	0.98	0.86-1.12	*52*	1.01	0.99-1.04
Fruit-Growing	*60*	0.93	0.75-1.15	*9*	0.98	0.70-1.37	*129*	0.90	0.77-1.06	*40*	0.87	0.58-1.34
Vegetables	*23*	1.22	0.88-1.70	*10*	0.25	0.02-2.96	*56*	0.95	0.75-1.21	*31*	1.00	0.93-1.06
Tobacco	*26*	1.33	0.90-1.95	*9*	0.79	0.36-1.74	*50*	0.91	0.68-1.22	*23*	1.06	0.91-1.24
Greenhouses	*7*	0.93	0.43-2.02	*4*	0.98	0.95-1.01	*15*	0.85	0.48-1.50	*8*	1.00	0.99-1.01

*ORs are calculated for the increment of 10 year for duration and for the increment of 10 animals/10 hectares, nE: number of cases exposed; *: numbers too limited (ne<3); All models adjusted for Age, Education Level, Tobacco Smoking, Alcohol Consumption, BMI. In the main analysis, the reference group consisted of non-agricultural participants.*

S3: Models with and without imputation of missing data for covariates (education, smoking, BMI, alcohol) adjusted on age -Illustrations

	Models with no imputation on covariates		Models with multiple imputation on the covariates		*% change in OR*
	OR	95% CI	OR	95% CI	
**Men**					
Pesticide use vs no use	1.14	0.85-1.53	1.14	0.91-1.43	*0%*
Work on a farm	1.81	0.92-3.57	1.59	0.98-2.58	*-12%*
Pesticide use on vines	2.03	0.98-4.24	1.94	1.15-3.28	*-4%*
**Women**					
Pesticide use vs no use	1.43	1.10-1.85	1.33	1.07-1.66	*-7%*
Work on a farm	1.58	0.94-2.66	1.16	0.79-1.71	*-27%*
Pesticide use on vines	2.02	0.98-4.16	1.56	0.90-2.69	*-23%*

Reference group: non-agricultural participants
